# Efficacy and Safety of Ivy Leaf Dry Extract EA 575 in Cough Management: An Updated Review of Clinical Evidence

**DOI:** 10.1055/a-2745-1130

**Published:** 2026-02-19

**Authors:** Inga Trompetter, Simon Braun

**Affiliations:** Engelhard Arzneimittel GmbH & Co. KG, Niederdorfelden, Germany

**Keywords:** Prospan, EA 575, cough, *Hedera helix*, Araliaceae, respiratory infection

## Abstract

EA 575, the ivy leaf dry extract of the herbal medicine Prospan, has been widely used for the management of cough associated with various respiratory conditions. Over the past decade, new evidence has emerged from interventional and non-interventional studies offering a more comprehensive understanding of its efficacy, safety, and tolerability. This review provides an updated synthesis of clinical evidence of EA 575 in pediatric and adult patients with respiratory disease. A total of 27 publications were included, covering 13 interventional trials, 13 non-interventional studies, and 1 meta-analysis. These studies collectively enrolled 102 239 patients, of whom 84 022 received EA 575 treatment. The findings consistently demonstrate that EA 575 improves cough symptoms and lung function, as shown by improvements in the Bronchitis Severity Score, spirometry, and body plethysmography, depending on the parameters that have been assessed. Moreover, the tolerability of EA 575 has been increasingly confirmed through large real-world evidence studies that report a low number of non-serious adverse events. These results reinforce the well-established role of EA 575 in the effective and safe treatment of cough in clinical practice.

**Abbreviations**
AE: Adverse event
BSS: Bronchitis Severity Score
DER: Drug-extract ratio
FEV _1_: Forced expiratory volume in 1 second
m/m: Mass/mass
MEF _75 – 25_: Maximal expiratory flow at 75 – 25% of the vital capacity
SAE: Serious adverse event
VC: Vital capacity
 

## Introduction

**Fig. 1:**
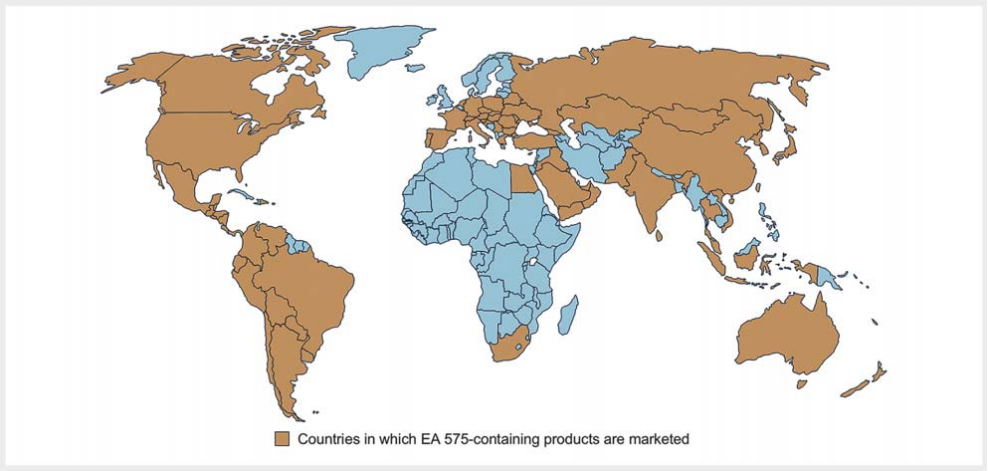
Global map showing countries where products containing EA 575 are currently marketed.

Cough is one of the most common symptoms managed in primary care 1 . It is a major contributor to productivity loss due to work absenteeism and poses a considerable economic burden on healthcare providers 2 , 3 , 4 . Acute cough is most frequently caused by acute respiratory tract infections, commonly referred to as the common cold, and acute bronchitis 5 . Infections are predominantly of viral origin, with viral etiology estimated at 83% for adults and 74% for children 6 , 7 . Consequently, and in light of the growing concern regarding bacterial resistance, antibiotic treatment is not indicated 8 , 9 . Extract preparations of dried leaves of common ivy ( *Hedera helix* L., Araliaceae) are widely used in over-the-counter herbal medicines for the management of cough 10 . EA 575 is a phytopharmaceutical extract of dried ivy leaves, standardized to a drug-extract ratio (DER) of 5 – 7.5 : 1, with 30% ethanol (m/m) as extraction solvent 11 . It is available on all continents except Antarctica ( Fig. 1 ), and various pharmaceutical formulations exist, including syrup, drops, and effervescent tablets 12 , 13 .

In 2020, the consensus guideline of the German Respiratory Society recommended EA 575 for managing cough during acute respiratory tract infections in adults, and this recommendation was reaffirmed in 2025 14 , 15 . The therapeutic evidence obtained with EA 575 cannot be generalized to other ivy-based extracts or combination products because the active ingredient content, and thus the effect, depends on many factors, such as the extraction method and production process 14 . Therefore, different ivy leaf dry extracts are not regarded as therapeutically interchangeable.

EA 575 is a multi-constituent plant preparation containing several classes of compounds with pharmaceutical activity, including saponins, flavonoids, and dicaffeoylquinic acids 16 , 17 . Therapeutic effects of EA 575 include bronchodilatory and secretolytic activities.

On a molecular level, these are mediated via inhibition of *β*_2_ -adrenergic receptor internalization in human airway smooth muscle cells and alveolar type II cells. This inhibition preserves receptor availability at the cell surface, thereby enhancing their responsiveness to the endogenous agonist adrenaline. The consequent upregulation of *β*_2_ -adrenergic receptor activation leads to increased cAMP production, which results in increased bronchodilation and secretion of surfactant 16 , 18 , 19 , 20 , 21 . EA 575 is the first phytopharmaceutical shown to induce biased *β*_2_ -adrenergic receptor activation 22 . Moreover, anti-inflammatory and antitussive activity of EA 575 has recently been demonstrated *in vivo* 23 .

Prospan, the only herbal medicine with EA 575, was introduced to the market in 1950 and has since been extensively studied through both clinical trials and post-marketing surveillance studies. A narrative review published in 2015 concluded that EA 575 is a valuable therapeutic option for managing both acute and chronic respiratory conditions in adult and pediatric patients 12 . Similarly, a narrative review from 2023 provided an overview of the clinical benefits of EA 575 in pediatric patients 24 .

As 2025 marks the 75th anniversary of Prospan and substantial clinical evidence has since been collected, this is a fitting occasion to publish an updated overview of the research findings. This narrative review builds on the 2015 review 12 and aims to provide a state-of-the-art summary of clinical data concerning the efficacy, safety, and tolerability of EA 575 in the management of cough.

## Methods

### Search strategy

MEDLINE (via the PubMed interface) was searched in July 2025 using the following search string: (“EA 575” OR “Prospan” OR “ivy leaf” OR “ivy leaves”). The search was then supplemented with additional publications known to the authors. Studies were considered eligible for inclusion if they assessed the efficacy, safety, and/or tolerability of EA 575 in patients presenting with respiratory diseases. All study designs, comparator types, and outcome measures were considered eligible for inclusion. No restrictions on age range, disease severity threshold, and article language were applied. Non-controlled studies published prior to 1992 were excluded, as the European Medicines Agency considers their methodology to be insufficient to show the efficacy of currently marketed products 25 .

## Results

### Overview of selected studies

**Fig. 2:**
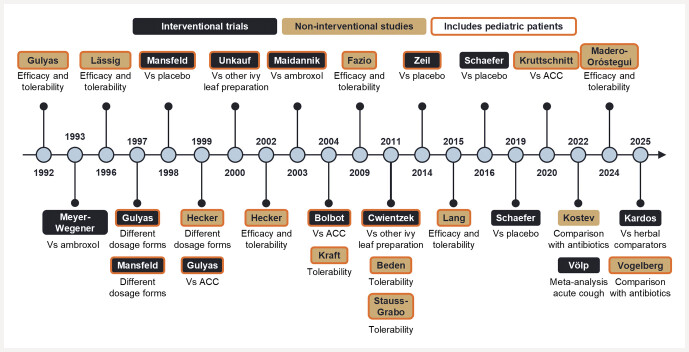
Timeline of clinical evidence for EA 575. The first authorʼs name is depicted in blue boxes (interventional trials) or gold boxes (non-interventional studies). Key assessments are indicated below each box. Studies exclusively or partially including pediatric patients are outlined with an orange border. ACC, acetylcysteine.

A total of 27 publications, published between 1992 and 2025, were included: 13 interventional trials, 13 non-interventional studies, and 1 meta-analysis ( Fig. 2 ). The studies were conducted across 15 countries (Argentina, Chile, Colombia, Czech Republic, Dominican Republic, Ecuador, Germany, Mexico, Paraguay, Peru, Slovenia, Switzerland, Ukraine, Uruguay, and Venezuela). Overall, 102,239 patients were enrolled in these studies, of whom 84,022 received EA 575 treatment.

### Interventional trials

An overview of the interventional trials is provided in Table 1 . These trials assessed the efficacy of EA 575 in patients with acute or chronic respiratory diseases, including bronchial asthma, acute cough, acute or chronic bronchitis, and chronic obstructive airways diseases. Seven trials were randomized double-blind 26 , 27 , 28 , 29 , 30 , 31 , 32 , two were randomized open-label 33 , 34 , one was quasi-randomized open-label 35 , and three were non-randomized 36 , 37 , 38 . Patient ages ranged from 0 to 86 years. The duration of the treatment was typically 7 – 10 days but could last for up to 30 days. In most trials, efficacy was assessed using pulmonary function tests to quantify respiratory function or the Bronchitis Severity Score (BSS) as a validated tool to examine therapeutic effects in common cold and acute bronchitis 39 . These evaluations were supplemented with additional measures, such as patient/parent- or clinician-rated global efficacy assessments and changes in specific symptoms including cough frequency/severity, expectoration, and respiratory pain.

**Table:** **Table 1** Summary of interventional trials with EA 575.

Reference	Design	Country	Number of patients (age range)	EA 575 dosage form(s)	Comparator	Duration	Therapeutic indication	Efficacy outcomes	Tolerability outcomes
**Placebo-controlled**									
Mansfeld et al. (1998) 27	Randomized, double-blind, crossover	Switzerland	28 (4 – 12 years)	Drops	Placebo	3 days	Bronchial asthma	**Primary endpoint** Body plethysmography: significant improvements in RAW with EA 575 vs. placebo **Secondary endpoints** Body plethysmography: significant improvements in ITGV with EA 575 vs. placebo Spirometry: clinically relevant, numerically greater improvements in FEV _1_ , FVC, and VC with EA 575 vs. placebo Patient/parent-rated assessment of cough symptoms: no differences between EA 575 and placebo in frequency of dose-metered aerosol use or intensity of cough and dyspnea	**Tolerability was evaluated** Patient/parent- and clinician-rated global assessment of tolerability: “very good” in all children in both groups SAE: Information is not apparent from the publication AE: Information is not apparent from the publication
Zeil et al. (2014) 28	Randomized, double-blind, crossover	Germany	30 (6 – 12 years)	Syrup	Placebo	28 – 30 days ^a^	Bronchial asthma	**Primary endpoint** Spirometry: no significant differences in relative change in FEV _1_ and MEF _75 – 25_ before bronchodilatation with EA 575 vs. placebo **Secondary endpoints** Significant improvements in the absolute change of MEF _75 – 25_ , MEF _25_ , and VC with EA 575 but not with placebo Body plethysmography (RAW), FeNO, EBC-pH, PAQLQ: no changes with EA 575 or placebo ACQ: decreased with placebo (p < 0.05) but not with EA 575	**Tolerability was evaluated** SAE: Information is not apparent from the publication AE: Information is not apparent from the publication
Schaefer et al. (2016) 29	Randomized, double-blind	Germany	181 (18 – 75 years)	Liquid	Placebo	7 days	Acute cough	**Primary endpoint** Cough severity (VAS over the whole treatment period) significantly reduced with EA 575 vs. placebo **Secondary endpoints** Cough severity (VAS over whole observation period, BSS, and VCD score) significantly reduced with EA 575 vs. placebo Patient/clinician-rated assessment of efficacy and condition: EA 575 significantly superior to placebo	**Tolerability was evaluated** SAE: 0 AE: 26 AEs were reported in 9 patients in the EA 575 group and in 12 in the placebo group; all AEs were non-serious and mild or moderate and not considered related to the study drug
Schaefer et al. (2019) 30	Randomized, double-blind	Germany	209 (18 – 73 years)	Liquid	Placebo	7 days	Acute bronchitis	**Primary endpoint** BSS (baseline to day 7): significant improvement with EA 575 vs. placebo **Secondary endpoints** BSS (day 3): significant improvement with EA 575 vs. placebo; no significant differences between the dosing schemes (5 mL t. i. d. vs. 7.5 mL b. i. d.) Cough severity (VAS, VCD score): significantly reduced with EA 575 vs. placebo Patient-rated assessment of global efficacy: EA 575 significantly superior to placebo	**Tolerability was evaluated** Patient-rated assessment of tolerability: “very well” or “good” in 99.3% with EA 575 and in 97.2% with placebo on day 7 SAE: 0 AE: 2928 patients reported AEs; 20 AEs were reported with EA 575 and 9 with placebo; all AEs were non-serious and mild or moderate Only 1 AE with suspected causal relationship (upper abdominal pain)
**Active comparator-controlled**									
Meyer-Wegener et al. (1993) 31	Randomized, double-blind	Germany	97 (25 – 70 years)	Drops	Ambroxol	4 weeks	Simple or obstructive chronic bronchitis	Spirometry: no significant differences in VC, FEV _1_ , PEF between groups Dry rale: no significant difference between groups Coughing, expectoration, and dyspnea: trend of greater improvement with EA 575 vs. ambroxol Patient-rated assessment of efficacy: “good” in 55.1% with EA 575 and 58.3% with ambroxol	**Tolerability was evaluated** Patient-rated assessment of tolerability: “good” in 87.8% with EA 575 and in 87.5% with ambroxol SAE: 0 AE: Information is not apparent from the publication AEs were reported in 7 patients in the EA 575 group (2 considered related to the study drug) versus 6 patients in the ambroxol group (3 considered related to the study drug)
Gulyas et al. (1997) 32	Randomized, double-blind, crossover	Germany	27 (10 – 16 years)	Syrup, drops	Different dosage forms	10 days	Chronic obstructive airways diseases	Spirometry: similar improvements in FEV _1_ , FVC, VC, and PEF with syrup and drops Body plethysmography: similar improvements in RAW, ITGV, and sRAW with syrup and drops	**Tolerability was evaluated** SAE: 0 AE: 0
Mansfeld et al. (1997) 35	Randomized, open-label, crossover	Switzerland	26 (5 – 11 years)	Suppositories, drops	Different dosage forms	3 days	Bronchial asthma	Patient/parent-rated assessment of cough symptoms: no clinically relevant changes or differences between groups in the intensity/frequency of cough and shortness of breath Spirometry: similar improvements in FEV _1_ and FVC with suppositories and drops Body plethysmography: similar decreases in RAW with suppositories and drops	**Tolerability was evaluated** Patient/parent- and clinician-rated global assessment of tolerability: “very good” in 25 children after both dosage forms SAE: Information is not apparent from the publication AE: Information is not apparent from the publication
Gulyas (1999) 37	Non-randomized ^b^	Germany	20 (9 – 15 years)	Syrup	Acetylcysteine	14 – 20 days	Chronic obstructive bronchitis	Spirometry: similar improvements in VC with EA 575 and acetylcysteine; greater improvements in FEV _1_ and PEF with EA 575 vs. acetylcysteine Body plethysmography: greater improvement in RAW with EA 575 vs. acetylcysteine	Not specified
Unkauf & Friederich (2000) 36	Quasi-randomized ^c^ , open-label	Germany	52 (0 – 12 years)	Syrup	Ivy leaf dry extract DER 3 – 6 : 1, 60% ethanol ^b^	10 days	Bronchitis	Clinician-rated assessment of cough symptoms: no significant differences in bronchitis, expectoration, or cough between groups Clinician-rated disease severity: similar improvements in bronchitis severity with EA 575 and DER 3 – 6 : 1 Clinician-rated assessment of bronchitis-related impairment: similar improvements, with non-inferiority of EA 575 vs. DER 3 – 6 : 1	**Tolerability was evaluated** SAE: 0 AE: 0
Maidannik et al. (2003) 38	Non-randomized ^b^ , open-label	Ukraine,	72 (0 – 15 years)	Syrup	Ambroxol	Acute disease: 7 – 10 days; chronic disease: 10 – 14 days	Acute and chronic respiratory diseases	Clinician-rated assessment of cough symptoms: reduction in shortness of breath frequency and productive cough with EA 575 and ambroxol; no significant differences between groups Patient/parent- and clinician-rated assessment of efficacy: “excellent” or “good” by 86.8% of patients/parents and 90.1% of clinicians External respiratory parameters (incl. VC, FVC, FEV _1_ , PEF, MEF _25_ , MEF _50_ ) exhibited greater improvements with EA 575 compared to ambroxol	**Tolerability was evaluated** SAE: Information is not apparent from the publication AE: Information is not apparent from the publication
Bolbot et al. (2004) 39	Non-randomized, open-label	Ukraine	50 (2 – 10 years)	Syrup	Acetylcysteine	7 – 10 days	Acute (obstructive and non-obstructive) bronchitis	Clinician-rated assessment of cough symptoms: cough frequency, sputum viscosity, shortness of breath, and respiratory pain normalized in a similar timeframe with EA 575 and acetylcysteine Spirometry: FEV _1_ , FVC, PEF, MEF _25_ , MEF _50_ , MEF _75_ , and AWSV _25 – 75_ showed significantly greater improvements with EA 575 vs. acetylcysteine after 5 days Clinician-rated global assessments of efficacy: “very good” or “good” in 96% with EA 575 and in 79.2% with acetylcysteine	**Tolerability was evaluated** Clinician-rated global assessments of tolerability: “very good” or “good” in 100% with EA 575 and in 76% with acetylcysteine SAE: Information is not apparent from the publication AE: Information is not apparent from the publication
Cwientzek et al. (2011) 33	Randomized, double-blind	Czech Republic	590 (2 – 86 years)	Drops ^b^	Ivy leaf soft extract DER 2.2 – 2.9 : 1, 50% ethanol/propylene glycol	7 days	Acute bronchitis	**Primary endpoint** BSS and BSS subscales: similar improvements in both groups, confirming non-inferiority of EA 575 vs. DER 2.2 – 2.9 : 1 **Secondary endpoints** Patient- and clinician-rated global assessments of efficacy: high and comparable between groups	**Tolerability was evaluated** Patient- and clinician-rated global assessments of tolerability: high and comparable between groups SAE: 0 AE: Information is not apparent from the publication AEs were reported in 2.7% of patients; most AEs were gastrointestinal and all were non-serious
Kardos et al. (2025) 34	Randomized, open-label	Germany	328 (18 – 75 years)	Drops	Ivy/thyme extract, thyme/primrose extract	7 days	Acute bronchitis	**Primary endpoint** BSS reduction: non-inferiority of EA 575 vs. comparators **Secondary endpoint** BSS reduction: superiority of EA 575 vs. ivy/thyme extract **Exploratory endpoints** BSS reduction from baseline to all post-baseline visits: significantly greater for EA 575 than ivy/thyme extract and thyme/primrose extract, respectively, for the 7-day treatment phase and over the further 7-day post-treatment observational period Cough severity (VAS): reduced with EA 575 vs. comparators Patient- and investigator-rated assessment of global efficacy: significantly better for EA 575 compared to ivy/thyme extract and thyme/primrose extract on days 7, 10, and 14	**Tolerability was evaluated** SAE: 0 AE: 13 Comparable AE frequency between groups (2.1% in EA 575 group, 1.1% in ivy/thyme comparator, and 9.5% in thyme/primrose comparator); AEs were non-serious 1 AE with possible relationship to EA 575 (diarrhea) Global patient- and investigator-rated tolerability was assessed as “fair”, “well”, or “very well” and comparable between groups
^a^ All patients received concomitant treatment with inhaled corticosteroids at a dosage equivalent of 400 µg/day of budesonide; ^b^ Randomization not specified; ^c^ Allocation rule by alternation; ACQ, Asthma Control Questionnaire; AE, adverse event; AWSV _27 – 75_ , average inhalation weight hour space velocity at 25 – 75% of the vital capacity; b. i. d., two times per day; BSS, Bronchitis Severity Score; DER, drug-extract ratio; EBC-pH, exhaled breath condensate pH; FeNO, fractional exhaled nitric oxide; FEV _1_ , forced expiratory volume in 1 second; FVC, forced vital capacity; ITGV, intrathoracic gas volume; MEF _25/50/75_ , maximal expiratory flow at 25/50/75% of the vital capacity; MEF _75 – 25_ , maximal expiratory flow at 75 – 25% of the vital capacity; PAQLQ, Standardized Paediatric Asthma Quality of Life Questionnaire; PEF, peak expiratory flow; (s)RAW, (specific) airway resistance; t. i. d., three times per day; SAE, serious adverse event; VAS, visual analog scale; VC, vital capacity; VCD, Verbal Category Descriptive									

Two randomized double-blind, placebo-controlled trials were conducted with EA 575 in children with bronchial asthma, and two randomized double-blind, placebo-controlled trials were performed in adults with acute respiratory tract infections 26 , 27 , 28 , 29 . One of the placebo-controlled trials in children with bronchial asthma reported significant improvements in body plethysmographic measurements and clinically relevant improvements in spirometric measurements with EA 575 26 . Although the other trial did not achieve statistical significance for its primary outcome measures, it did demonstrate significant improvements in the absolute change of spirometric measurements with EA 575 compared to placebo 27 , aligning with the previous findings from Mansfeld et al. No differences were found in the patient/parent-rated frequency and intensity of dyspnea and differentiated description of cough between EA 575 and placebo after three days of treatment 26 . More recent studies in adults with acute respiratory tract infections, including two double-blind, placebo-controlled trials that were statistically combined in a meta-analysis, showed a significant decrease in cough and bronchitis severity, as measured by the visual analog scale and BSS, respectively, after treatment with EA 575 compared to placebo. Furthermore, patient-reported assessment of efficacy and cough intensity and frequency improved significantly with EA 575 versus placebo 28 , 29 , 40 .

Two randomized trials (one double-blind and one open-label) compared the efficacy of different EA 575 dosage forms 31 , 34 . EA 575 drops versus syrup or suppositories demonstrated equivalent therapeutic efficacy in the management of chronic obstructive airways diseases and bronchial asthma in children, as determined by spirometry and body plethysmography 31 , 34 . Patient/parent-rated assessment of cough symptoms indicated no significant differences between groups, nor were there clinically relevant changes observed in the intensity or frequency of cough and dyspnea when comparing the different dosage forms 34 .

One randomized double-blind and one non-randomized open-label trial demonstrated comparable efficacy between EA 575, administered either as drops or syrup, and the synthetic mucolytic drug ambroxol in adults with simple or chronic bronchitis and children with acute and chronic respiratory diseases 30 , 37 . Remarkably, after 7 days of treatment with EA 575 syrup, normalization of external respiratory parameters was observed in 92.8% of the children, while such normalization could not be documented in the ambroxol group 37 .

Two non-randomized trials compared treatment with EA 575 syrup and the synthetic mucolytic drug acetylcysteine in children with acute and chronic obstructive bronchitis 36 , 38 . Both trials demonstrated greater improvements in forced expiratory volume in 1 second (FEV _1_ ) and peak expiratory flow when comparing EA 575 treatment with acetylcysteine 36 , 38 . Moreover, in Bolbot et al., forced vital capacity was significantly improved favoring EA 575, while comparable efficacy was found in the reduction in cough symptoms such as cough frequency, dyspnea, and respiratory pain 38 .

Finally, one randomized double-blind, one randomized open-label, and one quasi-randomized open-label trial in children and adults with acute bronchitis compared the efficacy of EA 575 with that of two other ivy leaf extracts (DER 3 – 6 : 1 or DER 2.2 – 2.9 : 1) or that of one ivy/thyme extract combination and one thyme/primrose extract combination 32 , 33 , 35 . These studies confirmed non-inferiority of EA 575 versus other herbal extracts in decreasing the severity of bronchitis (based on BSS and patient- or clinician-rated assessments) 32 , 33 , 35 .

One of these studies reported that EA 575 was non-inferior to thyme/primrose and even showed superiority compared to the ivy/thyme combination 33 .

The mean change from baseline in BSS was significantly greater with EA 575 than with ivy/thyme extract for all post-baseline visits (days 1, 2, 3, 4, 7, 10, and 14). This was the same when comparing EA 575 with thyme/primrose extract, except for one visit on day 7, which did not show statistical significance 33 .

EA 575 demonstrated a strong safety profile with a low number of adverse events (AEs) and high tolerability across various dosage forms 28 , 29 , 30 , 31 , 32 , 33 , 35 . Trials reporting on the global patient- and clinician-rated tolerability of EA 575 consistently demonstrated “good” to “very good” tolerability 26 , 29 , 30 , 32 , 33 , 34 , 38 . AEs were non-serious, ranging from mild to moderate, and were predominantly considered unrelated to the drug 28 , 29 , 30 , 32 , 33 . In three studies, AEs with a suspected causal relationship to EA 575 were documented for four patients in total 29 , 30 , 33 .

### Non-interventional studies

An overview of the non-interventional studies assessing the efficacy of EA 575 is provided in Table 2 . Nine of the studies were prospective (surveillance) studies 13 , 41 , 42 , 43 , 44 , 45 , 46 , 47 , 48 , two were retrospective cohort studies 8 , 49 , and two were retrospective chart reviews 50 , 51 . Patients with acute or chronic respiratory diseases, such as chronic obstructive airways diseases, bronchial asthma, acute and chronic (obstructive) bronchitis, and acute cough, were included. Patient ages ranged from 0 to 98 years. The therapeutic efficacy of EA 575 was evaluated in nine studies through the monitoring of symptom-specific changes and using clinician- or patient/parent-rated efficacy assessments. Two studies aimed at investigating the association between the prescription of EA 575 and the incidence of antibiotic use, repeated infections, and sick leave in adults. Two studies primarily assessed the tolerability and safety of EA 575.

**Table:** **Table 2** Summary of non-interventional studies with EA 575.

Reference	Design	Country/region	Number of patients (age range)	EA 575 dosage form (treatment duration)	Therapeutic indication	Efficacy outcomes	Tolerability outcomes
**With control group**							
Kruttschnitt et al. (2020) 48	Prospective, post-marketing Comparator: acetylcysteine	Switzerland	139 (6 – 95 years)	Syrup	Acute bronchitis	Patient/parent- and clinician-rated assessment of cough symptoms: reductions in cough intensity, chest pain, and cough-related sleep disturbance No significant difference between EA 575 and acetylcysteine in improvement and efficacy assessments, except for dyspnea, which showed a higher improvement with EA 575 BSS: continuous reduction in score	**Tolerability was evaluated** Patient- and clinician-rated global assessment of tolerability: “very good” or “good” by 98.3% of patients/parents and by 99.1% of clinicians SAE: 0 AE: 2
Kostev et al. (2022) 50	Retrospective, cohort study Comparator: antibiotic drug	Germany	14 068 (≥ 18 years)	Any dosage form	Common cold: viral infection of unspecified site, acute nasopharyngitis (common cold), acute upper respiratory infections of multiple and unspecified sites, acute bronchitis, non-specified bronchitis, or cough	EA 575 associated with significantly lower odds of a new antibiotic prescription vs. antibiotic prescription 4 – 30 days after the diagnosis date (OR: 0.83; 95% CI: 0.72 – 0.96) and 31 – 365 days after the diagnosis date (OR: 0.44; 95% CI: 0.40 – 0.48) EA 575 associated with lower odds of bacterial infections 4 – 30 days after the diagnosis date (OR: 0.45; 95% CI: 0.16 – 1.31) In patients with at ≥ 1 day of sick leave, EA 575 significantly associated with lower odds of sick leave of > 7 days vs. antibiotic prescription (OR: 0.81; 95% CI: 0.73 – 0.90); including patients with 0 days of sick leave, EA 575 significantly associated with higher odds of sick leave of ≥ 7 days vs. antibiotic prescription (OR: 1.46; 95% CI: 1.35 – 1.58) EA 575 associated with significantly lower odds of new cough diagnosis vs. antibiotic prescription (OR: 0.91, 95% CI: 0.85 – 0.98)	Not specified
Vogelberg et al. (2025) 9	Retrospective, cohort study Comparator: antibiotic drug	Germany	20 780 (0 – 17 years)	Not specified	Common cold: acute URTI of multiple and unspecified sites, acute bronchitis, bronchitis, not specified as acute or chronic, cough, acute nasopharyngitis, or viral infection of unspecified site	EA 575 associated with significantly lower odds of subsequent antibiotic prescriptions vs. antibiotic prescription 4 – 30 days after the diagnosis date (OR: 0.56; 95% CI: 0.49 – 0.64) and 31 – 365 days after the diagnosis date (OR: 0.58; 95% CI: 0.54 – 0.62) EA 575 associated with significantly lower odds of bacterial infections 4 – 30 days after the diagnosis date (OR: 0.67; 95% CI: 0.45 – 0.99)	Not specified
**Without control group**							
Gulyas & Lämmlein (1992) 49	Prospective	Germany	26 (4 – 10 years)	Syrup (4 weeks)	Chronic obstructive bronchitis	Spirometry, dry rale and patient-rated dyspnea, coughing fits, and expectoration: significant improvements Clinician-rated assessment of global effectiveness: “good” or “excellent” in 65.4% of patients	**Tolerability was evaluated** Clinician-rated assessment of tolerability: “excellent” in 92.3% of patients SAE: 0 AE: 0
Lässig et al. (1996) 42	Prospective, surveillance	Germany	113 (6 – 15 years)	Syrup	Recurrent obstructive respiratory diseases	Clinician-rated assessment of cough symptoms: significant improvements in frequency of cough and expectoration Spirometry: significant improvements in FEV _1_ , FVC, PEF, MEF _25_ , and MEF _50_ Clinician-rated assessments of improvement: 85.7% of children were “cured” or “significantly improved”	**Tolerability was evaluated** Clinician-rated assessments of tolerability: “very good” or “good” in 98.2% SAE: 0 AE: 0
Hecker et al. (1999) 43 ·	Prospective, post-marketing surveillance	Germany	248 (0 – 79 years)	Syrup (age 0 – 9 years) or effervescent tablets (age ≥ 4 years)	Inflammatory and/or obstructive respiratory diseases	Clinician-rated assessment of cough symptoms: “cured” or “improved” cough in 87.5%, expectoration in 91.3%, shortness of breath in 57.7%, and respiratory pain in 60.9% Clinician-rated global assessments of efficacy: “very good” or “good” efficacy in 86%	**Tolerability was evaluated** Clinician-rated global assessments of tolerability: “very good” or “good” in 98% SAE: 0 AE: 1 1 AE was reported (allergic exanthema)
Hecker et al. (2002) 44	Prospective, post-marketing surveillance	Germany	1350 (4 – 98 years)	Effervescent tablets	Chronic bronchitis	Clinician-rated assessment of cough symptoms: “cured” or “improved” cough in 92.2%, expectoration in 94.2%, shortness of breath in 83.1%, and respiratory pain in > 80% Clinician-rated global assessment of efficacy: “very good” or “good” in 91%	**Tolerability was evaluated** SAE: 0 AE: 6 AEs were reported in 6 patients (0.4%); 3 discontinued treatment (0.2%)
Kraft (2004) 51	Retrospective chart review	Germany	52 478 (0 – 12 years)	Syrup	Symptomatic respiratory diseases	Not specified	**Tolerability was evaluated** SAE: 0 AE: 115 AEs were reported in 0.22% of patients (diarrhea, 0.1%; enteritis, 0.04%; allergic exanthema/urticaria, 0.04%; vomiting, 0.02%)
Fazio et al. (2009) 45	Prospective, post-marketing surveillance	Latin America	9657 (0 – 98 years)	Syrup (7 days)	Acute or chronic bronchitis	Clinician-rated assessment of cough symptoms: improvement/healing of cough in 93.4%, expectoration in 92.9%, dyspnea in 91.2%, and pain in 90.8%	**Tolerability was evaluated** Clinician-rated global assessment of tolerability: “very good” or “good” in 96.6% SAE: 0 AE: 198 AEs were reported in 2.1%; all non-severe; mostly gastrointestinal Discontinuation: 4.0%; 1.9% due to improvement/healing
Beden et al. (2011) 46	Prospective, post-marketing, surveillance	Slovenia	193 (2 – 14 years)	Syrup (7 days)	Acute respiratory tract infection	Clinician-rated assessment of cough symptoms: complete or partial improvement in coughing (95.3%) and expectoration (93.0%); most children with difficulty breathing and pain experienced complete improvement Patient- and clinician-rated assessments of improvement: 93.7% had “complete” or “partial” improvement	**Tolerability was evaluated** Treatment tolerability: “very good” or “good” in 97.9% SAE: 0 AE: 1 1 AE (urticaria) was reported; 3 children discontinued treatment (1.7%)
Stauss-Grabo et al. (2011) 47	Prospective, post-marketing surveillance	Germany	331 (11 – 85 years)	Tablets (≥ 7 days)	Colds with cough, chronic inflammatory bronchial diseases	Not specified	**Tolerability was evaluated** Patient- and clinician-rated global assessment of tolerability: “very good” or “good” by 96.4% of patients and by 98.5% of clinicians AE: 0 AE: 1 1 AE (nausea) was reported, which was non-serious
Lang et al. (2015) 14	Prospective, post-marketing surveillance	Germany	1066 (6 – 12 years)	Syrup, drops, liquid, effervescent tablets, and lozenges (approximately 7 days)	Acute bronchitis	Patient- and clinician-rated assessment of cough symptoms: patients reported 42.1 – 58.1% decreases in cough intensity, chest pain, shortness of breath, sleep problems, expectoration, and cough attacks; clinicians reported ~ 50% decreases in intensity of cough attacks, expectoration/sputum, dyspnea, and breath sounds BSS: 79.3% improvement Therapeutic equivalence between different dosage forms Patient- and clinician-rated global assessments of efficacy: “very good” or “good” by 91.2% of patients and 93.2% of clinicians	**Tolerability was evaluated** Patient- and clinician-rated global assessments of tolerability: “very good” or “good” by 95.4% of patients and 96.7% of clinicians SAE: 0 AE: 1010 AEs were reported in 10 patients (0.9%); 7 gastrointestinal, 2 allergic, 1 other
Madero-Oróstegui et al. (2024) 52	Retrospective chart review	Colombia	80 (2 – 12 years)	Syrup	Acute bronchitis	Resolved cough in 50.0% and 76.2% of children after 7 and 14 days, respectively BSS reduced at each follow-up Nocturnal awakenings due to coughing decreased from 3 at baseline to 0 Patient-rated satisfaction with EA 575: “very satisfied” or “satisfied” in 98.8% of patients Patient- and clinician-rated assessment of treatment success: “complete recovery” or “major improvement” by 97.5% of patients and 100% of clinicians	**Tolerability was evaluated** SAE: 0 AE: 0
AE, adverse event; BSS, Bronchitis Severity Score; CI, confidence interval; FEV _1_ , forced expiratory volume in 1 second; FVC, forced vital capacity; MEF _25/50_ , maximal expiratory flow at 25/50% of the vital capacity; OR, odds ratio; SAE, serious adverse event; PEF, peak expiratory flow; URTI, upper respiratory tract infection							

One prospective study compared the efficacy and tolerability of EA 575 with that of acetylcysteine in patients with acute bronchitis 47 . Results showed continuous reduction in BSS and improvement of cough symptoms and sleep disturbance after 7 days of treatment with the ivy leaf dry extract. EA 575 and acetylcysteine showed comparable efficacies for all assessments, except for dyspnea, which showed greater improvement with EA 575 treatment 47 .

Two retrospective cohort studies, one in adults and one in children with common cold diseases, evaluated the association between EA 575 or antibiotic prescriptions and several respiratory disease parameters 8 , 49 . In both studies, EA 575 was associated with significantly lower odds of a new antibiotic prescription within the month and year following the index date (date of diagnosis) compared to matched patients who received an initial antibiotic. Furthermore, the use of EA 575 was significantly associated with lower odds of bacterial infections in children in the month after the index date 8 and lower odds of a new cough diagnosis in adults in the year after the index date 49 . In adult patients who took at least one day of sick leave, EA 575 was significantly related to lower odds of at least a 7-day-long sick leave compared to those who received an initial antibiotic prescription 49 .

Two prospective studies reported clinically relevant improvements in spirometric measurements after EA 575 treatment of children with chronic obstructive bronchitis or recurrent obstructive respiratory diseases 41 , 48 . Additionally, other studies (one prospective and one retrospective) demonstrated significant improvements in BSS in children with acute bronchitis 13 , 51 , with the study from Lang et al. showing therapeutic equivalence among the five evaluated dosage forms (syrup, drops, liquid, effervescent tablets, and lozenges) 13 . Various patient- and clinician-rated cough symptoms, including cough frequency and intensity, expectoration, shortness of breath, respiratory pain, and cough attacks, were significantly improved with EA 575 treatment in patients with recurrent obstructive respiratory diseases, inflammatory and/or obstructive respiratory diseases, acute and chronic bronchitis, and acute respiratory tract infections 13 , 41 , 42 , 43 , 44 , 45 . Furthermore, the large-scale surveillance study conducted by Lang et al., involving > 1000 children diagnosed with acute bronchitis, demonstrated a 58.1% reduction in sleep problems, as reported by patients or their parents, after seven days of EA 575 treatment as a consequence of cough symptom reduction 13 . Similar results were reported in a retrospective chart review from 2024 that reported a median decrease in nocturnal awakenings from three to zero after seven days of treatment 51 .

Across the non-interventional studies, the patient- and clinician-rated global efficacy of EA 575 for the treatment of inflammatory and/or obstructive respiratory diseases and of acute and chronic bronchitis was reported as “good” or “very good” by 91.2% of the patients and 65.4 – 93.2% of the clinicians 13 , 42 , 43 , 48 . Moreover, assessments of treatment success by patients and clinicians revealed complete or partial improvement in 85.7 – 100% of children with recurrent obstructive respiratory diseases, acute respiratory tract infections, or acute bronchitis 41 , 45 , 51 . Madero-Oróstegui et al. further reported that 98.8% of children with acute bronchitis were either “satisfied” or “very satisfied” with EA 575 treatment 51 .

Safety and tolerability data for EA 575 in non-interventional studies were similar to those in interventional trials. Global assessment of the tolerability of EA 575 was rated “good” or “very good” by ≥ 96.6% of the clinicians and ≥ 95.4% of the patients 13 , 41 , 42 , 44 , 45 , 46 , 47 , 48 . Overall, EA 575 treatment resulted in a low number of AEs or adverse drug reactions and included non-serious allergic exanthema/urticaria, diarrhea, enteritis, vomiting, and nausea 13 , 41 , 42 , 43 , 44 , 45 , 46 , 47 , 48 , 50 , 51 .

## Discussion

This review presents an update on the efficacy and safety results of ivy leaf dry extract EA 575 for the management of cough. In addition to the studies included in the previous review in 2015 12 , three interventional trials 28 , 29 , 33 and five additional non-interventional studies have been included 8 , 13 , 47 , 49 , 51 . These studies provide enhanced insights into the efficacy, safety, and tolerability of EA 575, formulated as syrup, liquid, drops, effervescent tablets, or lozenges for treating acute bronchitis, acute cough, and the common cold 8 , 13 , 28 , 29 , 47 , 49 , 51 . Furthermore, additional data have become available, including information on the evaluation of EA 575 compared to two herbal extract combinations 33 and acetylcysteine 47 . New evidence also explored the association between EA 575 prescriptions and broader health outcomes, such as sleep problems, antibiotic usage, duration of sick leave, and recurrent infections 8 , 13 , 47 , 49 , 51 .

Evaluating the efficacy of therapeutic interventions for acute respiratory diseases poses significant challenges, as the infections are self-limiting and typically resolve spontaneously within seven to ten days 52 , 53 . In a study by Cwientzek et al., Hedelix was found to be non-inferior to EA 575 in treating mild bronchitis. However, caution should be taken when interpreting the clinical significance of this finding, as patients exhibited mild symptoms likely to resolve within the 7-day treatment period. Since both groups fully recovered by the studyʼs end, the absence of a difference may reflect the natural course of the illness rather than true therapeutic non-inferiority 32 . Spontaneous recovery often masks actual treatment effects, making it challenging to demonstrate efficacy beyond placebo in trials involving short, self-limiting conditions such as acute cough 54 . Frequent assessment visits during the early stages of the disease are essential to detect meaningful differences between treatment arms. Notably, Völp et al. showed a statistically significant early onset of efficacy, with BSS improvements observed as early as two days after initiating treatment with EA 575 compared to placebo 40 . Supporting these results, Kardos et al. reported that the minimal clinically important difference of the BSS change from baseline was achieved as early as day 2 33 . The rapid onset of action observed with EA 575 is particularly relevant for patients with a common cold, who often seek prompt relief to resume everyday life, family responsibilities, and work.

Objective measurements such as spirometry and body plethysmography offer valuable insights into pulmonary function changes. EA 575 demonstrated favorable effects on lung function parameters in several studies comparing different dosage forms 31 , 34 , placebo-controlled trials 26 , 27 , and non-interventional studies 41 , 48 . Notably, Zeil et al. showed significant improvements in the secondary endpoints maximal expiratory flow at 75 – 25% of the vital capacity (VC), maximal expiratory flow at 25% of the VC (MEF _25_ ), and VC in children with bronchial asthma who were treated with EA 575 compared to placebo. However, no significant improvement in the primary endpoint FEV _1_ was observed 27 . The lack of FEV _1_ response highlights an ongoing controversy regarding its use as the principal parameter for classifying bronchial asthma severity in pediatric populations. This controversy stems from anatomical differences in children. Their relatively large airway diameter in proportion to lung volume can result in normal FEV _1_ values despite having been diagnosed with asthma 55 , 56 , 57 . Consequently, MEF _75 – 25_ and VC will possibly establish themselves as more reliable and sensitive parameters for the assessment of bronchial asthma in children 58 , 59 .

Comparative trials with active treatments that evaluated lung parameters demonstrated that EA 575 has yielded favorable outcomes in three out of four studies. Two studies comparing EA 575 with acetylcysteine in children with acute or chronic obstructive bronchitis reported significant improvements in lung function in favor of the ivy leaf dry extract 36 , 38 . Comparisons with ambroxol have been less consistent. One study found no significant differences in external respiratory parameters between the two treatments in patients with bronchitis 30 , while another reported greater improvement with EA 575 compared to ambroxol in children with acute and chronic respiratory diseases 37 . These findings suggest that EA 575 may offer greater improvement in lung function compared to acetylcysteine or ambroxol, potentially due to an additional bronchospasmolytic effect 37 , 38 . Further comparative trials are required to quantitatively assess whether these differences translate into clinical superiority. Zeil et al. and Mansfeld et al. also observed improvement in lung function parameters, though their trials involved comparisons to placebo 26 , 27 . These effects may be attributable to the bronchodilatory activity of EA 575, which has been mechanistically supported by various cell culture and *ex vivo* experiments 19 , 20 . This bronchodilatory effect is clinically noteworthy, as significant reversibility of airway obstruction following bronchodilator medication use has long been recognized as a main hallmark of asthma 60 . Consequently, classic bronchodilatory agents are defined by their ability to improve lung function parameters.

Additional efficacy endpoints in the comparative trials with active treatments included evaluations of global efficacy and cough-related symptoms. Overall, global efficacy of EA 575 was rated as “good” or “very good” and was better in comparison to that of ivy/thyme and thyme/primrose extracts and–according to one study–acetylcysteine 13 , 32 , 33 , 35 , 37 , 38 , 42 , 43 , 48 . Over the years, a whole range of cough-related symptoms have been investigated in addition to cough, including cough-related sleep disturbance. Reduced cough symptoms during treatment with EA 575 also led to improvements in related symptoms such as cough-related sleep disturbance, thereby contributing to an enhancement in the patientʼs quality of life 13 , 47 , 51 . Especially non-interventional studies reported a wide range of subjective symptomatic improvements with EA 575 treatment 13 , 41 , 42 , 43 , 44 , 45 , 47 , 51 .

Overall, efficacy was frequently in favor of EA 575 when compared with acetylcysteine, ambroxol, and other ivy leaf extracts and demonstrated statistical superiority compared to ivy/thyme extract 30 , 32 , 33 , 36 , 37 , 38 , 47 . Future studies incorporating additional objective parameters, such as device-mediated quantification of coughing events, would be a welcome addition. These data could offer further insights to complement and strengthen the existing evidence supporting the therapeutic benefits of EA 575 61 .

In comparative analyses of EA 575 and antibiotic therapy for the treatment of viral infection of unspecified site, acute nasopharyngitis, acute upper respiratory infections of multiple and unspecified sites, acute bronchitis, non-specified bronchitis, and cough, EA 575 was associated with significantly reduced odds of subsequent antibiotic prescriptions in both pediatric and adult populations 8 , 49 .

The value of real-world evidence studies in informing healthcare decision-making is increasingly acknowledged. Randomized controlled trials remain the methodological gold standard for evaluating the efficacy and safety of interventions and for assessing causality, as they minimize confounding variables. However, their external validity can be limited due to highly selective inclusion and exclusion criteria 62 , 63 . In contrast, real-world evidence studies encompass broader, more heterogeneous patient populations, thereby enabling the assessment of therapeutic interventions under routine clinical conditions 63 , 64 . Real-world data can address evidence gaps that are not captured by non-interventional trials and reinforce existing findings, particularly by enabling the collection of large datasets on safety events, heterogenous study populations with different concomitant medications and diseases, and different ethnic groups 62 . Over the past decade, an increasing number of real-world evidence studies have focused on EA 575. These studies, characterized by large sample sizes, consistently demonstrated a low number of AEs, supporting a favorable tolerability and safety profile of EA 575 8 , 49 , 50 , 51 . A low incidence of AEs was found in both children and adults. This is in line with the conclusions from the previous reviews by Lang et al. and Seifert et al. 12 , 24 . Differences in safety between children and adults have not been systematically assessed within the context of clinical trials, but safety data are mostly contributed to by post-marketing surveillance studies with children ( Table 2 ). Moreover, the European Union herbal monograph on *Hedera helix* of the Committee on Herbal Medicinal Products concludes that ivy preparations are well tolerated in all oral formulations, based on long clinical use and literature, without serious unwanted pharmacodynamic actions on any organ system 25 . However, while the safety data benefit from robust sample sizes, efficacy outcomes should be interpreted with caution, as they rely primarily on subjective measures, namely patient- or clinician-rated global assessments of efficacy. Across the studies, these evaluations suggested clinical benefits with EA 575 8 , 49 , 50 , 51 . Although these findings reflect a generally positive perception of EA 575′s efficacy, real-world evidence should be viewed as complementary to, rather than a substitute for, data derived from rigorously conducted interventional trials. When considered alongside data from the other interventional trials and non-interventional studies, the cumulative evidence provides compelling support for the efficacy, safety, and tolerability of EA 575 in patients of all ages.

However, interpretation of the overall evidence presents several challenges. A systematic review published in 2021 included multiple studies investigating EA 575 65 . These studies ranged from interventional trials to non-interventional studies and were assessed as having a low 28 , 29 , moderate 32 , or high 13 , 46 risk of bias. The main sources of bias identified in this systematic review were uncontrolled confounding, selective reporting of results, and subjective outcome measurements without blinding 65 . Several studies included in the current review were sponsored by the manufacturers of the investigated products. While industry-funded studies may carry a risk of reporting favorable outcomes, it is important to recognize that publicly funded research for already marketed products is mostly unavailable. The market authorization holder is often the only party positioned to support further investigation. Therefore, it is important to include industry-funded studies as data sources, especially for post-marketing data and real-world evidence 66 . The current review identified important considerations related to evaluating the efficacy of EA 575, particularly in pediatric populations, where placebo-controlled studies are generally limited in this therapeutic area and in the context of acute and chronic respiratory diseases–areas where robust data remain relatively scarce 67 . Accordingly, children should receive special attention in future research projects, and modern measurement methods such as device-based cough monitoring should be considered.

In general, the studies discussed in this review show how much the standards of clinical studies have improved in recent years. Older studies are informative, but some lack detailed information, e.g., on the study design, confounder adjustment, or the severity of AEs. Evidence of the effect based on these older data has been verified in recent years in modern clinical trials that fully meet all standards. Overall, this gives a typical picture of an herbal extract that has been continuously well studied over the years in accordance with the applicable standards.

## Conclusions

Since the previous EA 575 review, eight new studies have been included, expanding the dataset to encompass results from 15 different countries. In total, 84 022 patients received EA 575 treatment in clinical studies. The findings of this review suggest that EA 575 is characterized by a favorable safety profile and very good tolerability in patients with cough, as supported by interventional trials and large real-world evidence studies. Interventional and non-interventional studies from the last decade further support the proven clinical benefits of EA 575 in managing respiratory airways diseases. Future research directions should address the need for well-designed, randomized, placebo-controlled trials that evaluate the efficacy of EA 575 during acute respiratory tract infections in children. Furthermore, continuous and objective measurement of cough frequency or patterns by AI-powered monitoring devices may be used in future studies to monitor disease progression and treatment effects in greater detail.
